# Optogenetic and chemogenetic insights into the food addiction hypothesis

**DOI:** 10.3389/fnbeh.2014.00057

**Published:** 2014-02-28

**Authors:** Michael J. Krashes, Alexxai V. Kravitz

**Affiliations:** Diabetes, Endocrinology, and Obesity Branch, National Institute of Diabetes and Digestive and Kidney Diseases, National Institutes of HealthBethesda, MD, USA

**Keywords:** obesity, addiction, optogenetics, food, feeding, arcuate, striatum

## Abstract

Obesity is clinically diagnosed by a simple formula based on the weight and height of a person (body mass index), but is associated with a host of other behavioral symptoms that are likely neurological in origin. In recent years, many scientists have asked whether similar behavioral and cognitive changes occur in drug addiction and obesity, lending many to discuss the potential for “food addiction”. Advances in understanding the circuitry underlying both feeding behaviors and drug addiction may allow us to consider this question from the viewpoint of neural circuits, to complement behavioral perspectives. Here, we review advances in understanding of these circuits and use them to consider whether drawing comparisons to drug addiction is helpful for understanding certain forms of obesity.

Drug addiction is a chronic, relapsing disorder that is characterized by physical signs such as tolerance and withdrawal, as well as emotional and behavioral symptoms such as sensations of craving and compulsive reward-seeking. Tolerance describes a phenomenon in which higher doses of a drug are required to achieve an effect, while withdrawal signs describe a range of physiological and emotional consequences that occur when an addict stops taking a drug. The behavioral changes associated with drug addiction can be broadly grouped into three main categories (Koob and Volkow, 2010). First, drugs and associated cues exert strong effects on reinforcement processes, driving drug-directed behavior to become compulsive. Second, drug addiction is accompanied by impaired inhibitory control processes, which normally act as the brakes on behavior. Finally, drug addiction is complemented by negative emotional states such as anxiety and depression, which can serve as triggers to drive further drug use. Indeed, drug-abstinent humans and animals are most vulnerable to relapse during periods of emotional stress or hardship (Epstein et al., 2006; Koob, 2008; Erb, 2010; Sinha et al., 2011). These three classes of symptoms may reflect alterations in distinct circuitry, which work together to facilitate drug use in addicted individuals. We will describe recent optogenetic and chemogenetic studies that have provided hypothetical maps of what this circuitry might be.

The term “food addiction” was introduced into the literature in the 1950s (Randolph, 1956), but there were few published studies on this topic in the subsequent 60 years. Instead, a large number of researchers addressed drug addiction during this time (Figure 1). This has changed in very recent years, during which a small but growing number of researchers have begun investigating food addiction. Modern researchers are in an ideal position to investigate this link, as the United States and many other countries have become entrenched in an obesity epidemic that must be addressed (Centers for Disease Control, 2013), and societal acceptance of “food addiction” is commonplace, as evidenced by the large number of support groups for over-eating, many of them based on the 12-step framework developed to address drug and alcohol dependence (Weiner, 1998; Russell-Mayhew et al., 2010). Indeed, several measures of substance use (particularly cigarette smoking) in the US have been on the decline in recent decades, while the prevalence of obesity has risen steadily (Centers for Disease Control, 2013).

**Figure 1:**
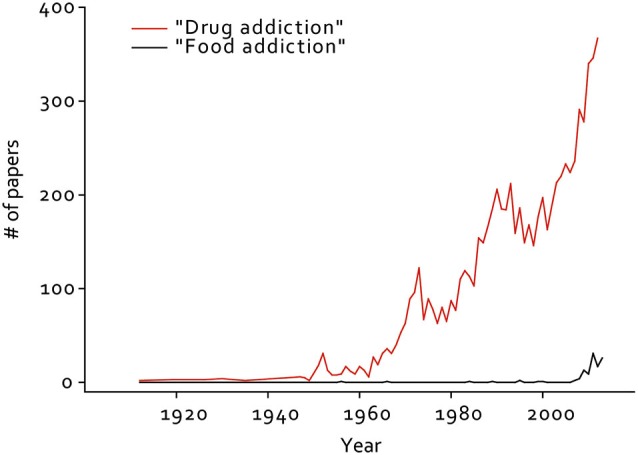
**Number of papers published per year from 1912–2012 containing the term “drug addiction” or “food addiction” in the title or abstract.** Results from a Pubmed search on 11/08/13, using tools from the Neuroscience Information Framework (NIF). RIID:nif-0000-25673

Like drug addiction, obesity is a complex disorder with multiple causes and symptoms. For example, a small number of obese individuals have monogenic receptor mutations (such as in the leptin and melanocortin receptors) that cause extreme weight gain (Farooqi and O’Rahilly, 2008). However, the majority of obesity that has developed in the past 30 years is not believed to be the result of monogenic mutations, but rather changes in our food supply and lifestyles during this time (Farooqi and O’Rahilly, 2008). The behavioral signs and symptoms that are associated with this obesity can be loosely mapped to the same categories as drug addiction: compulsive overconsumption, difficulty controlling the intake of food, and the emergence of negative emotional states such as anxiety and depression (Kenny, 2011a; Sharma and Fulton, 2013; Sinha and Jastreboff, 2013; Volkow et al., 2013). Therefore, it is possible that the circuit changes underlying these processes in obesity are similar to those that occur during drug addiction. It is worth noting, however, that like drug addiction, specific obese individuals often exhibit subsets of these dysfunctions, such that an individual is likely to exhibit different specific symptoms, and alterations in circuitry. In addition, feeding depends on homeostatic feeding circuitry which is critical for survival, a distinct difference from drug addiction.

Conceptually, feeding has often been regarded as the product of two independent networks that integrate and control food intake, hunger and hedonic pleasure (Kenny, 2011b). In addition to reward circuitry that likely contributes to both drug addiction and obesity, a homeostatic system also regulates food intake based on caloric need by circulating blood borne factors such as glucose, free fatty acids, leptin, ghrelin and insulin (Myers and Olson, 2012; Adan, 2013; Hellström, 2013). These engage hypothalamic and brainstem circuits to promote or blunt feeding responses, thus contributing to normal energy balance. This is one way in which obesity differs from drug addiction, as obesity may reflect alterations in homeostatic feeding circuitry, in addition to changes in reward circuitry. Importantly, novel tools have been developed that allow neuroscientists to manipulate circuits with unprecedented precision and control (Fenno et al., 2011; Rogan and Roth, 2011; Tye and Deisseroth, 2012). In this review, we outline recent research on the circuitry underlying both feeding and drug addiction, and discuss the degree to which analysis of this circuitry can shed new light on the similarities and differences between obesity and drug addiction.

## Circuitry mediating homeostatic feeding

Studying the mechanisms of homeostatic food intake is challenging due to slow temporal kinetics of the parameters mediating the switch between hunger and satiety. Hormones need to be released from peripheral tissues, travel to the brain and signal nutrient-sensing neurons to direct food-seeking and consumption behavior. These prolonged changes in energy deficit considerably hamper the examination of the contributing relationships between deprivation-sensitive sensory systems and the downstream brain circuits they engage. To side-step this difficulty, manipulations of molecularly circumscribed nutrient-sensing neurons can be used to prove the central control of feeding. Once identified, the afferent and efferent pathways modulating both hunger and satiety can be further analyzed in detail (Sternson, 2013).

The arcuate nucleus (ARC) of the hypothalamus constitutes a variety of diverse cell-types that are ideally situated to integrate blood-borne signals released from peripheral tissues, as the ARC rests at the base of the brain adjacent to the third ventricle and median eminence. Specifically, two distinct ARC subpopulations, orexigenic agouti-related protein (AGRP) and anorexigenic proopiomelanocortin (POMC) neurons have been substantially linked to alterations in food intake. Both heterogeneous subtypes are conversely stimulated and inhibited by the fat-derived hormone leptin (Myers and Olson, 2012) and the energy signals glucose (Claret et al., 2007; Fioramonti et al., 2007) and insulin (Konner et al., 2007; Hill et al., 2010). Moreover, AGRP neurons are directly activated by the hunger-promoting gut-derived hormone ghrelin (Cowley et al., 2003; van den Top et al., 2004). Further bolstering their respective contributions to eating, pharmacological injections into the brain of the neuromodulators released by AGRP neurons, the peptides AGRP and neuropeptide Y (NPY) escalate feeding (Semjonous et al., 2009), while α-melanocyte stimulating hormone (α-MSH) and adrenocorticotrophic hormone (ACTH), released from POMC neurons, attenuate food intake (Poggioli et al., 1986).

Optogenetic or chemogenetic (Aponte et al., 2011; Krashes et al., 2011, 2013; Atasoy et al., 2012) activation of AGRP neurons is sufficient to rapidly elicit voracious food intake, even in calorically replete animals, linking activation of these neurons to the perception of hunger and subsequent feeding. Importantly, the degree of consumption is dependent on both the number of excitable neurons and stimulation frequency (Aponte et al., 2011). Chronic activation of these neurons and the resulting hyperphagia and reduced energy expenditure leads to marked weight gain, accompanied by increased fat stores (Krashes et al., 2011). Furthermore, the neuromediators released by AGRP neurons drive biphasic feeding episodes with GABA and/or NPY promoting acute food intake while the peptide AGRP orchestrates food consumption over a delayed, chronic scale (Atasoy et al., 2012; Krashes et al., 2013). Interestingly, animals with acutely stimulated AGRP neurons during a normal resting period, in the absence of food, display intense, unabated locomotor activity that is completely reversed in the presence of food, strongly suggesting a foraging role for these neurons (Krashes et al., 2011). Furthermore, remote AGRP-induction significantly increases an animal’s willingness to work for food in a classic nosepoke assay (Krashes et al., 2011).

To investigate the downstream functional contributions of AGRP neurons on feeding, long-range axon projections were photostimulated and resulting food intake was assessed. Selective terminal-field activation in the paraventricular (PVN) hypothalamus evoked feeding in a similar magnitude to direct somatic AGRP activation, implicating a crucial role for neurons in this brain site in directing appetite signaling (Atasoy et al., 2012). To definitively demonstrate this, two forms of chemogenetic inhibition were used to silence the majority of PVN neurons, resulting in escalated *ad lib* food intake and motivation to work for food. Furthermore, elegant occlusion studies whereby AGRP afferents to the PVN and downstream PVN neurons marked by a mouse oxytocin (OXT) promoter fragment were co-transduced with channelrhodopsin-2 (ChR2) and simultaneously photostimulated, completely reversing the AgRP→PVN-evoked increase in food intake. Finally, by applying combinatorial opto- and chemogenetic manipulations with pharmacology, alternative downstream circuits of AGRP neurons were implicated in eliciting feeding behavior. Recently, it was revealed that AGRP axonal projections to the bed nucleus of the stria terminalis (BNST), lateral hypothalamus (LH) or paraventricular thalamus (PVT), in addition to the PVN, are sufficient to drive feeding (Betley et al., 2013; need to add this ref PMID: 24315102). Importantly, distinct AGRP axonal projections that target different anatomical brain regions originate from specific subpopulations, whereby a “one-to-one” axon collateral configuration for AGRP neurons governs downstream connectivity (Betley et al., 2013).

Conversely to experiments testing AGRP sufficiency, tools used to acutely suppress AGRP neurons revealed their necessity in feeding (Krashes et al., 2011), which parallels the hypophagic response in animals following conditional ablation of these cells (Gropp et al., 2005; Luquet et al., 2005). This neural ablation approach led to the identification of an anorexia circuit in the parabrachial nucleus (PBN; Wu et al., 2009), which receives inhibitory input from AGRP neurons (Atasoy et al., 2012) and critical excitatory input from the nucleus of the solitary tract (NTS), which in turn is activated via serotonergic projections from the raphe magnus and obscurus (Wu et al., 2012). Notably, acutely abrogating glutamatergic signaling from the PBN increases food intake, implicating the importance of excitatory tone from this anatomical region in guiding feeding behavior (Wu et al., 2012). To further demonstrate the PBN has key regulator of appetite, a novel circuit, marked by calcitonin gene-related peptide-expressing neurons, projecting to the central nucleus of the amygdala has been shown to mediate feeding responses (Carter et al., 2013).

Direct POMC manipulations have the opposite effect on appetite as chronic optogenetic and chemogenetic (Aponte et al., 2011; Zhan et al., 2013) activation of this ARC population decreases food intake. This effect requires intact melanocortin signaling, as mice with constitutively-suppressed melanocortin-4 receptors failed to exhibit this hypophagic response (Aponte et al., 2011). Furthermore, acute stimulation of POMC neurons in the NTS attenuates food intake with fast-acting kinetics (hours) vs. the slower-acting ARC-expressing POMC neurons (days) (Zhan et al., 2013). However, only the latter are necessary for mediating satiety as acute ablation of ARC-expressing POMC neurons causes hyperphagia and obesity (Zhan et al., 2013). Further studies investigating both downstream targets and upstream circuits regulating these AGRP and POMC neurons are required to unravel a functional, wiring diagram modulating appetite control.

While this elegant work has elucidated much of the important circuitry that controls homeostatic feeding under natural conditions, it is not clear whether plasticity in this circuitry contributes to behavioral changes associated with obesity, nor whether targeting this circuitry would be effective for long term weight loss (Halford and Harrold, 2012; Alvarez-Castro et al., 2013; Hellström, 2013). Although obese people eat more, it is not clear whether obese people experience stronger perceptions of hunger or reduced perceptions of satiety, beyond the physiological need to eat more to sustain a larger body size (French et al., 2014). Future studies may investigate the intrinsic firing of these neural populations, as well as plasticity mechanisms among these neurons to address this. Intriguingly, a recent study demonstrated genetic perturbation of AgRP neural activity from development or postnatal ablation of these neurons enhanced exploratory behavior and intensified responses to cocaine, indicating that alterations in these neurons can contribute to behavioral plasticity associated with other brain regions (Dietrich et al., 2012). Chronic manipulations of these circuits may address the extent to which these circuits are altered in obesity, as well as their therapeutic potential for long term weight loss.

## Beyond homeostatic feeding

Evidence for the potential of animals to engage in non-homeostatic feeding was demonstrated in classic electrical stimulation and lesion experiments of the lateral hypothalamus (Delgado and Anand, 1953; Margules and Olds, 1962; Wise, 1974; Markou and Frank, 1987), which can cause rodents to eat far beyond homeostatic need. Recent work has elucidated that this likely depended on inhibitory projections from the BNST, marked by Vesicluar GABA transporter (VGAT) to the LH (Jennings et al., 2013). Optogenetic stimulation of these GABAergic projections evoked robust feeding in sated mice and time spent in a designated food zone, while inhibition of these projections diminished feeding in hungry mice. Interestingly, these bidirectional optogenetic perturbations revealed that this GABA^*BNST*^→Glutamate^*LH*^ circuit had significant influence on motivational valence. Manipulating this pathway in an orexigenic direction evoked appetitive, rewarding responses as assessed using real-time place preference and self-stimulation assays, while manipulation in an anorexigenic direction elicited aversive responses (Jennings et al., 2013). Remarkably, the same study demonstrated both necessity and sufficiency for a glutamatergic sub-population of neurons in the LH marked by the expression of *Vglut2* (glutamate transporter 2; Jennings et al., 2013). While manipulations of the LH can produce a range of effects on motivated behavior (including complete cessation of feeding) (Hoebel, 1971; Wise, 1974), optogenetic stimulation of these VGAT^*BNST*^→VGLUT^*LH*^ projections or direct optogenetic inhibition of VGLUT^*LH*^ neurons specifically produced voracious feeding behavior, suggesting that explicit hypothalamic afferent projections or populations of LH neurons likely support different aspects of feeding behavior. This point has been noted for decades (Wise, 1974), however the emergence of novel tools and techniques have allowed investigators to understand more specifically which neural populations and projections support different aspects of feeding behavior.

## Craving and compulsive consumption of food rewards

Craving is a core feature of drug addiction, which is believed to underlie the compulsive consumption of drugs of abuse (Koob and Volkow, 2010). Obese people often experience craving for food as well, and the circuitry that correlates with craving in obesity appears to be similar to that in drug addiction (Avena et al., 2008; Jastreboff et al., 2013). This includes dopaminergic circuitry, and adaptations in these structures are likely to be responsible for heightened craving in both drug addiction and obesity (Volkow et al., 2002; Wang et al., 2002). The largest populations of dopaminergic neurons reside in the midbrain, in the substantia nigra pars compacta (SNc) and the ventral tegmental area (VTA). Optogenetic activation of midbrain dopaminergic neurons in mice facilitated positive reinforcement during food-seeking behavior in an operant task (Adamantidis et al., 2011) in addition to a more generalized place preference test (Tsai et al., 2009). Similar positive reinforcing properties, as assessed by intracranial self-stimulation, of these neurons were observed in rats (Witten et al., 2011). GABAergic neurons of the VTA directly inhibit dopaminergic VTA cells and optogenetic activation of the former is sufficient to drive conditioned place aversion as well as consummatory behavior (Tan et al., 2012; van Zessen et al., 2012). Intriguingly, in the conditions used in the Adamantidis study, stimulation of dopaminergic terminals alone was not reinforcing, although it facilitated positive reinforcement of food-maintained behavior (Adamantidis et al., 2011). This suggests that a special relationship may exist between reinforcement in feeding contexts, such that animals have a lower threshold for learning about food-related information than other information.

The reinforcing actions of dopamine likely depend on dopamine dependent plasticity onto or within striatal neurons that receive input from midbrain dopaminergic structures. These are mainly medium spiny neurons that express either the dopamine D1 or D2 receptor, known as direct pathway (dMSNs) or indirect pathway medium spiny neurons (iMSNs), respectively (Gerfen et al., 1990). A model for how these striatal populations control behavior was introduced in the late 1980s, and is sometimes referred to as the “classic model” of basal ganglia circuitry (Albin et al., 1989). Based largely on anatomical studies, these authors hypothesized that activation of dMSNs facilitated motor output, whereas activation of iMSNs inhibited motor output. Explicit tests of this model have supported it, demonstrating that direct pathway promotes movement, whereas the indirect pathway inhibits movement (Sano et al., 2003; Durieux et al., 2009; Kravitz et al., 2010).

However, just as dopamine can promote both reinforcement and movement, dMSNs and iMSNs also exhibit an opposing influence over reinforcement, which may suggest physiological links between movement and reinforcement (Kravitz and Kreitzer, 2012). The dopamine D1 receptor is an excitatory Gs coupled receptor, and thus dopamine can excite dMSNs through this receptor (Planert et al., 2013), which may be integral to the reinforcing properties of dopamine. Indeed, optogenetic stimulation of dMSNs is sufficient to drive operant reinforcement in mice (Kravitz et al., 2012), and modulation of dMSNs activity can modulate the reinforcing properties of cocaine and amphetamine (Lobo et al., 2010; Ferguson et al., 2011) and natural rewards (Hikida et al., 2010) in a manner consistent with the effects of direct dMSN stimulation. The dopamine D2 receptor is an inhibitory Gi coupled receptor, and thus dopamine inhibits iMSNs through this receptor (Planert et al., 2013). Optogenetic activation of D2 receptor expressing iMSNs promotes aversion (Kravitz et al., 2012), and also reduces preference (Lobo et al., 2010), and self-administration of cocaine (Bock et al., 2013). Consistent with this, chemogenetic inhibition of these neurons enhances the rewarding properties of amphetamine and cocaine (Ferguson et al., 2011; Bock et al., 2013). Similarly, when food deprived rats were given a choice between palatable food (chocolate biscuits) and their normal chow, the D1 agonist SKF 38393 increased their preference for the palatable food, while the D2 agonist quinpirole reduced it (Cooper and Al-Naser, 2006). In this way, dopamine release can promote reinforcement through two independent basal ganglia circuits. Dopamine may promote reinforcement through activating dMSNs and activity through the direct pathway, as well as through inhibiting iMSNs and activity through the indirect pathway (Kravitz and Kreitzer, 2012).

While dopamine release is normally reduced as animals learn reinforcement relationships, sucrose binging can repeatedly evoke high levels of dopamine release, repeatedly providing a reinforcement signal following behaviors directed at these foods (Rada et al., 2005; Hoebel et al., 2009). Whether repeated dopamine release occurs with high fat or other palatable diets is not known. The repeated dopamine release during sucrose binging may be similar to what happens with addictive drugs, which also continue to stimulate dopaminergic function through pharmacological actions, irrespective of how well the animal has learned the association between a behavior and drug delivery (Di Chiara and Imperato, 1988). Therefore, as animals consume such diets, dopamine mediated reinforcement processes may occur at repeated and super-physiological levels. Indeed, obesity has been associated with enhanced activity in areas of the brain that process salience and reward in response to visual food stimuli (Rothemund et al., 2007; Stoeckel et al., 2008; Jastreboff et al., 2013), although other studies have reported opposing findings on this point (Stice et al., 2010). Importantly, especially when considering similarities and differences between drug addiction and sucrose addiction, different subset of striatal neurons are activated when animals self-administer cocaine vs. food or water, indicating that different “functional units” throughout the basal ganglia may subserve behaviors directed at drug vs. food reinforcers (Carelli et al., 2000). Despite this functional organization, it is possible that similar pathological changes in dopamine mediated reinforcement processes may contribute to compulsive consumption in subset of striatal units that subserve both food and drug addiction. The above studies elucidated pathways that can modulate the reinforcing properties of drugs of abuse, and suggest that these pathways may be altered in drug addiction. However, this is only one component of addiction, which is a complex disease involving many brain circuits. In addition to drug-mediated reinforcement through basal ganglia circuits described above, other circuits mediate impairments in inhibitory control, and the emergence of negative emotional states. While the above have better elucidated the role of the dopaminergic system in mediating reinforcement, it is important to note that not all reinforcement is addiction. For example, the vast majority of individuals that experience drugs of abuse do not become addicted, despite finding the drugs reinforcing. Therefore, other circuitry changes are likely involved in drug addiction, such as those underlying deficits in inhibitory control over behavior, and the emergence of negative emotional states.

## Impairments in inhibitory control

Drug addiction is accompanied by impairments in medial prefrontal and orbitofrontal cortical function, and resulting deficits in executive control over behavior (Koob and Volkow, 2010; Volkow et al., 2013). In animals, a recent study demonstrated that prolonged cocaine self-administration decreases cellular excitability of pre-frontal cortical neurons, potentially pointing to a mechanism for how repeated cocaine use impairs frontal circuitry (Chen et al., 2013). To directly test the role of PFC neurons in compulsive cocaine seeking, these authors optogenetically stimulated and inhibited these neurons, which attenuated or increased compulsive cocaine seeking, respectively (Chen et al., 2013). Although in a different behavioral paradigm, different results were reported with cue-induced reinstatement of cocaine seeking, where inhibition of this structure impaired cue-induced reinstatement of cocaine seeking (Stefanik et al., 2013). This difference indicates that prefrontal impairments in the human studies may not be reflective of simple decreases in prefrontal activity, but rather more specific changes in distinct prefrontal circuits in ways that enhance relapse potential. Indeed, optogenetic stimulation studies demonstrate that specific PFC neurons projecting to the largely serotonergic dorsal raphe promote active swimming in a forced swim test, while activation of all PFC neurons do not (Warden et al., 2012). It is possible that different pre-frontal cortical circuits facilitate defined aspects of drug-related behavior, and as such, may be revealed by different behavioral paradigms.

Similar cortical deficits may also be associated with obesity. The diet industry is sustained by the inability of humans to control their eating without external interventions. There is increasing evidence that obesity is associated with impairments in cognitive function, including deficits in executive function, working memory, and attention (Gunstad et al., 2007; Bruehl et al., 2009; Mirowsky, 2011). These functions are served by cortical circuitry, which exerts a “top-down” control over subcortical brain circuits discussed above. Brain imaging studies have revealed a number of structural abnormalities associated with obesity, such as decreases in gray matter volume and metabolic activity in frontal regions of obese people, likely contributing to impairments in the ability to inhibit eating (Le et al., 2006; Pannacciulli et al., 2006; Volkow et al., 2009; Smucny et al., 2012; Van den Eynde et al., 2012).

One situation in which humans often find themselves attempting to exert inhibitory control is during dieting. A dieting human is attempting to maintain a calorically-deficient state, while resisting both reinforcement mechanisms (outlined above) and emotional stressors (outlined below). An animal model of this is stress-induced reinstatement of food seeking. In this paradigm, animals are trained to lever-press for food, after which this is extinguished but can be reinstated with stressors, including the pharmacological stress mimic yohimbine (and α2-adrenergic antagonist). Optogenetic inhibition of the medial PFC during yohimbine treatment impaired this reinstatement, similar to reports with cue induced reinstatement of cocaine, suggesting that similar processes may underlie both results (Calu et al., 2013; Stefanik et al., 2013). Again, this indicates that cortical dysfunctions associated with obesity are likely not simple changes in overall activity, but rather the specific activity of specific prefrontal projections. Indeed, a Fos activation study in both food and stress reinstatement paradigms revealed that activated prefrontal neurons exhibit unique synaptic alterations, relative to non-activated neurons (Cifani et al., 2012). A focal point for future research will investigate the terminal projections of these pre-frontal cortical neurons, which have been shown to send axons to reward centers such as the VTA and accumbens core. Such studies will allow us to address the extent to which prefrontal dysfunctions are similar or different between obesity and drug addiction.

## Negative emotional states

Negative emotional states such as anxiety and depression can be strong triggers that drive drug use in addicts. Addicts are most vulnerable to relapse during periods of stress or emotional distress, and drug use can promote stressful and emotionally distressing situations (Koob, 2008). Similar patterns can occur with over-eating associated with obesity, causing researchers to question whether similar circuitry underlies stress evoked drug and food addiction (Parylak et al., 2011; Sinha and Jastreboff, 2013). For example, periods of stress are often associated with the consumption of highly palatable foods, giving rise to the terms “comfort foods” and “emotional eating”. In addition, obese animals exhibit higher levels of anxiety and depression, suggesting that these foods themselves contribute to a cycle in which these negative emotional states contribute to further eating (Yamada et al., 2011; Sharma and Fulton, 2013).

Multiple brain systems regulate negative emotional states, including the dopamine system. Altered dopamine signaling has been heavily implicated in obesity as both obese humans and rodents have lower levels of striatal dopamine D2 receptor (D2R) availability compared with lean people and animals (Wang et al., 2001; Johnson and Kenny, 2010). In addition, polymorphisms in the D2 receptor gene (*Drd2*) have been linked to obesity and multiple forms of drug addiction (Blum et al., 1990; Noble et al., 1993; Stice et al., 2008; Chen et al., 2012). Interestingly, although deficits in D2R availability have also been linked to addiction to cocaine, alcohol, opiates, and nicotine, these addictions are not associated with weight gain. This suggests that the effects of D2 receptor impairments are not linked to weight gain *per se*, but to the overlapping behavioral changes that accompany both obesity and drug addiction. One hypothesis for how reduced D2R function may contribute to behavioral changes associated with both obesity and drug addiction is that animals consume more to compensate for blunted dopaminergic responses as a result of decreased receptor levels (Wang et al., 2002; Stice et al., 2008). In other words, animals require higher levels of dopaminergic stimulation to get the same effect as an animal with a full complement of dopamine receptors. This can be accomplished through pharmacological means, as all drugs of abuse result in dopamine release in the striatum (Di Chiara and Imperato, 1988). Alternatively, it may be accomplished through the consumption of palatable foods, such as food that are high in sugar and fat.

Reduced D2R function may be predicted to elevate activity in iMSNs, as D2R is a Gi coupled receptor. Therefore, it is possible that obese individuals consume foods that over-stimulate dopamine release to inhibit these overactive iMSNs and escape from pervasive negative emotional states. Consistent with this hypothesis, animals that express ChR2 in iMSNs exhibit aversion to stimulation of these cells (Kravitz et al., 2012). When examined in the context of cocaine reward, optogenetic stimulation also impairs (Lobo et al., 2010; Bock et al., 2013), while chemogenetic inhibition of these neurons enhanced cocaine directed behaviors (Ferguson et al., 2011; Bock et al., 2013). Consistent with these findings, increases in the rewarding properties of amphetamine were detected when these neurons were ablated (Durieux et al., 2009). Together, these findings suggest that reductions in D2 expression may produce a pervasive negative emotional state, and that animals will seek super-physiological dopamine release to escape from this state.

In addition to dopamine receptors, alterations in dopamine producing neurons in the VTA may contribute to the emergence of negative emotional states. Through their inputs to the VTA, efferents emanating from the laterodorsal tegmentum and the lateral habenula elicit positive and negative states in mice, respectively (Lammel et al., 2012; Stamatakis and Stuber, 2012). Selective inhibition of VTA DA neurons induced depression-like phenotypes, as assessed via tail-suspension and forced-swim tests, in addition to anhedonia, quantified through a sucrose preference assay (Tye et al., 2013). To demonstrate bidirectional control of these neurons and their sufficiency in mediating these behaviors, the authors showed that temporally sparse phasic photoactivation of VTA DA neurons rescues stress-induced depression-like phenotypes (Tye et al., 2013). To investigate susceptibility vs. resilience to social-stress-induced behavioral irregularities, it was reported that optogenetic induction of phasic, but not tonic, firing in VTA DA neurons of mice undergoing a subthreshold social-defeat paradigm promoted social avoidance and decreased sucrose preference, two independent readouts of depression (Chaudhury et al., 2013). Dopamine neurons in the VTA have long been known to encode consummatory reward and reward-predictive cues (Bayer and Glimcher, 2005; Pan et al., 2005; Roesch et al., 2007; Schultz, 2007). Electrophysiological studies have also linked VTA DA neurons to stress and negative states (Anstrom et al., 2009; Wang and Tsien, 2011; Cohen et al., 2012) highlighting the complexity of dopaminergic signaling.

Finally, in humans, the amygdala has been linked to both anxiety-disorders (Etkin et al., 2009) and craving (Childress et al., 1999; Wrase et al., 2008), in addition to a host of other emotional processes. Several optogenetic studies have dissected amygdala circuits in connection with a wide array of behaviors from those related to anxiety (Tye et al., 2011; Felix-Ortiz et al., 2013; Kim et al., 2013) or fear (Ciocchi et al., 2010; Haubensak et al., 2010; Johansen et al., 2010) as well as those related to reward-seeking (Stuber et al., 2010; Britt et al., 2012). While electrophysiological studies demonstrate that amygdala neurons encode both positive and negative motivational valence (Paton et al., 2006; Shabel and Janak, 2009), there have not yet been studies genetically identifying the neural encoding dynamics of the partially non-overlapping populations of neurons that do so. While the neural correlates of negative emotional states associated with obesity are not fully understood, examination of synaptic and cellular alterations in these circuits may be a promising place to look.

## Conclusion

In recent years, the drug addiction paradigm has been applied to the neural circuits mediating behaviors associated with obesity. This perspective has sparked important insights, while still recognizing that obesity has important differences from drug addiction. Primarily, food is necessary for survival, which makes parsing the adaptive and maladaptive components of feedings a challenge when thinking of potential therapies, as obese people cannot develop strategies to avoid food altogether, as a drug addict might towards drugs of abuse. Given the ability of feeding behaviors to be both necessary for survival and harmful in excess, understanding the neural circuits related to food addiction calls for tools of utmost precision, such as manipulations facilitated by optogenetic and chemogenetic approaches.

## Conflict of interest statement

The authors declare that the research was conducted in the absence of any commercial or financial relationships that could be construed as a potential conflict of interest.
